# Perceptions of antimicrobial use and resistance among pet owners in Chile: A cross-sectional One Health survey

**DOI:** 10.14202/vetworld.2025.2450-2459

**Published:** 2025-08-26

**Authors:** Nicolás Galarce, Ailén Dumont-Viollaz, José Longa, Leslye Camila del Río, Andrea Núñez, Byron Guzmán-Marín, Pamela Thomson

**Affiliations:** 1 Department of Animal Preventive Medicine, Faculty of Veterinary and Animal Sciences, University of Chile, Santiago 8820808, Chile; 2 School of Veterinary Medicine, Faculty of Life Sciences, Andres Bello University, Santiago, 8370251, Chile; 3 One Health Institute, Faculty of Life Sciences, Andres Bello University, Santiago, 8370251, Chile; 4 Doctoral Program in Conservation Medicine. Andres Bello University, Santiago, 8370251, Chile; 5 School of Sociology. Faculty of Law and Social Sciences, Silva Henríquez Catholic University, Santiago, 8330225, Chile; 6 School of Veterinary Medicine, Faculty of Agricultural and Forestry Sciences, Catholic University of Maule, Curicó, 3340000, Chile; 7 Faculty of Engineering and Sciences, Universidad Adolfo Ibáñez, Santiago, 7550344, Chile

**Keywords:** antibiotics, antimicrobial resistance, cats, Chile, dogs, One Health, perceptions, pet owners

## Abstract

**Background and Aim:**

Antimicrobial resistance (AMR) presents a critical global health threat, compromising the efficacy of treatments across human, animal, and environmental health domains. While efforts have predominantly focused on livestock and human medicine, the role of pet owners remains underexplored, despite their direct involvement in antimicrobial administration and influence on veterinary decisions. This study aimed to assess perceptions, knowledge, and practices regarding antimicrobial use and resistance among pet owners in Chile, providing a baseline to inform future education and policy initiatives under a One Health framework.

**Materials and Methods:**

A cross-sectional survey targeting adult dog and cat owners was conducted from May 19 to 21, 2023, in Santiago, Chile, during a large public pet-focused event. Using a validated questionnaire, data were collected on demographics, pet characteristics, knowledge, attitudes, and practices related to antibiotics and AMR. A total of 378 valid responses were analyzed. Descriptive statistics and Chi-square tests were applied to examine associations, particularly between education level and reported practices.

**Results:**

Most respondents were female (74.4%) with professional degrees (64%) and aged between 21 and 50 years. Dogs were more commonly owned (73.8%) than cats (26.2%). While 86.2% reported their pet had received antibiotics, only 68.2% recalled veterinarians confirming proper administration understanding. A strong majority agreed that antibiotics should only be used for bacterial infections and opposed acquiring them without a prescription. Notably, 92.4% stated that they would stop treatment early if improvement was observed. Approximately 52% had used human antibiotics for pets, and 38% were open to substituting prescribed veterinary antibiotics with human ones. No significant association was found between education level and antibiotic use behaviors (p > 0.05).

**Conclusion:**

Findings reveal important gaps in owner understanding and adherence to antimicrobial guidelines, particularly regarding treatment completion and human antibiotic use in pets. Strengthening veterinarian-owner communication, promoting species-specific antibiotic use, and enhancing awareness of AMR’s broader implications are essential. These insights support targeted educational efforts and integration of pet owner perspectives into national AMR strategies to advance One Health goals.

## INTRODUCTION

Antimicrobial resistance (AMR) represents a significant global health threat because it compromises the effectiveness of antimicrobial agents used to treat infections in humans, animals, and plants [1–3]. Without intervention, AMR infections could result in up to 10 million deaths annually and cause a projected global economic loss of USD 100 trillion by 2050 [4].

In response, many countries adopted the Global Action Plan (GAP) in 2015 to combat AMR [5]. The plan was developed collaboratively by the World Health Organization, the Food and Agriculture Organization, and the World Organization for Animal Health [6]. In 2022, the United Nations Environment Program also joined the global AMR initiative, reinforcing the need for a multidisciplinary and collaborative approach [7]. The GAP outlines core objectives, including raising awareness through education and promoting the responsible use of antimicrobials in both human and veterinary sectors. These objectives target not only healthcare workers, veterinarians, and plant health professionals but also aim to engage the broader community [6].

Despite global efforts to control AMR, focusing primarily on livestock, humans, and food production chains [8, 9], companion animals remain largely overlooked. This is notable given that they account for approximately 30% of veterinary antimicrobial use and are often treated with off-label human medications [10–13]. Studies on veterinary prescribing practices have identified frequent deviations from antimicrobial stewardship guidelines. Common issues include the use of critically important antibiotics as first-line treatments, a high frequency of empirical prescriptions, and the use of antibiotics without confirmed bacterial infections [14–16].

In contrast, limited research has focused on the perceptions of pet owners, despite their key role in administering antimicrobials and influencing veterinary treatment decisions [17]. While pet owners generally trust veterinarians and follow instructions, their understanding of AMR-related risks varies significantly across different contexts [18–20].

Although some progress has been made – such as increased public awareness of AMR in the UK [21, 22] – studies by Smith *et al*. [22] and Scarborough *et al*. [23] in the UK and Australia reveal persistent gaps in understanding, particularly regarding zoonotic transmission. For example, Scarborough *et al*. [23] reported that while most respondents agreed veterinarians should prescribe antibiotics only, when necessary, few recognized the broader human and environmental implications. In contrast, Candellone *et al*. [24] found that 84% of participants understood AMR, and 96% viewed it as a serious issue affecting both human and animal health. Similarly, Taylor and Scallan Walter [19] observed that 62% of pet owners acknowledged that antibiotic use in pets could contribute to AMR, and over 90% supported prudent prescribing practices. However, fewer respondents recognized AMR as a threat to animal health (44.4%) or acknowledged the possibility of interspecies transmission (29.1%).

Interestingly, Candellone *et al*. [24] found no significant relationship between education level and AMR knowledge or concern. Such knowledge gaps may hinder broader recognition of the public health risks posed by inappropriate antimicrobial use in pets, including the potential transmission of resistant bacteria to humans [19, 25]. Therefore, addressing AMR effectively requires public education for pet owners and enhanced training for veterinarians in antimicrobial stewardship [22].

In 2017, Chile adopted a One Health strategy with the launch of its National Plan Against AMR [26]. The plan set specific targets for antimicrobial use in companion animals, emphasizing infection prevention, control, and usage monitoring [27]. This initiative involves collaboration with multiple organizations, including the Intersectoral Roundtable for the Control of AMR in Companion Animals, which is coordinated by the Chilean Veterinary Medical Association [28]. Initial analyses of prescribing patterns revealed that the most commonly prescribed first-choice antibiotics for pets in Chile were amoxicillin-clavulanic acid, enrofloxacin, and doxycycline. Notably, many of these prescriptions were empirical and not based on laboratory testing, despite being classified as critically important antimicrobials in clinical settings [29].

Despite global efforts to combat AMR, research and policy initiatives have primarily focused on livestock, human health, and food production systems. Companion animals – particularly dogs and cats – have received comparatively little attention, even though they represent a substantial proportion of veterinary antibiotic use and frequently receive medications based on empirical judgment. Notably, pet owners are directly involved in administering antimicrobials and can influence prescription practices through expectations or misunderstandings, yet their perceptions, knowledge, and behaviors remain understudied in many regions. Most of the available studies on pet owners’ understanding of AMR originate from high-income countries such as the UK, Italy, Australia, and the United States. These studies show inconsistent levels of awareness, especially regarding zoonotic transmission and the implications of using human antibiotics in animals. However, there is a lack of comparable data from Latin American contexts, particularly from Chile, where national One Health strategies have been implemented but pet owner engagement remains poorly characterized. Moreover, few studies have examined the relationship between owners’ demographic profiles (e.g., education level, health sector affiliation) and their antibiotic use behaviors. This represents a critical gap in achieving effective antimicrobial stewardship in companion animal medicine.

To address this gap, the present study aimed to assess the perceptions, knowledge, and self-reported practices related to antimicrobial use and resistance among traditional pet owners in Chile. By conducting a cross-sectional survey at a large-scale public pet-focused event in Santiago, this research sought to: (1) establish a baseline of owner awareness regarding prudent antibiotic use and AMR; (2) examine how demographic factors influence antimicrobial-related attitudes and behaviors; and (3) identify actionable targets for educational, veterinary, and policy interventions. The findings are intended to inform Chile’s ongoing national AMR efforts and contribute to broader One Health strategies by incorporating the often-overlooked perspectives of companion animal caregivers.

## MATERIALS AND METHODS

### Ethical approval and Informed consent

The study complied with the ethical principles outlined in the Declaration of Helsinki for research involving human participants and was approved by the Scientific Ethics Committee of the Central-South Macrozone at the University of Santo Tomás (Approval code: CEC-CS-UST-129-21). Before participation, all respondents provided informed consent, and the confidentiality of their data was ensured.

### Study period and location

A cross-sectional survey was conducted between May 19 and 21, 2023, in Santiago, Chile. The study targeted adult Chilean dog and cat owners, who were invited to participate voluntarily.

### Development and validation of survey instruments

A minimum sample size of 300 completed surveys was calculated to ensure statistical validity, based on a 95% confidence level and a 5% margin of error. The initial version of the survey was developed collaboratively by two veterinary physicians specializing in companion animal medicine and one with expertise in microbiology. The draft was subsequently reviewed and validated by a sociologist. Revisions were made to clarify Module 3, particularly regarding the identification of participants affiliated with the healthcare sector and to enhance overall comprehension. The revised version was pre-tested by 10 veterinarians, who reported no further suggestions for improvement. The final survey instrument was approved by the sociologist before deployment.

### Participant recruitment and data collection

Participants were recruited in person at a large-scale pet-focused public event in Santiago, attended by approximately 8,000 individuals (https://www.expomascotasyanimales.com). A convenience sampling method was employed. Inclusion criteria required participants to be at least 18 years old and to be the primary caretaker (tutor) of at least one dog or cat. The study’s objectives, data usage protocols, and confidentiality safeguards were explained to all respondents. Informed consent and survey instruments were distributed and collected in physical (paper) format.

### Survey content and structure

The survey comprised four structured modules:

- Module 1: Demographic information (age, sex, education level, and professional affiliation with the health sector).
- Module 2: Pet characteristics (species, age, sex, and breed).
- Module 3: Knowledge and attitudes related to antibiotic use and AMR, assessed using a 5-point Likert scale ranging from “strongly agree” to “strongly disagree”.
- Module 4: Self-reported practices concerning veterinary antibiotic use and communication with veterinarians.

### Data management and statistical analysis

Survey responses were digitized using Microsoft Excel (Microsoft Corporation, Redmond, WA, USA) and organized by module. The dataset was cleaned and coded before statistical evaluation. All analyses were performed using RStudio (version 2023.06.0421, R Core Team, Vienna, Austria). Frequency distributions were calculated for each variable. Demographic variables (age, sex, education level, and health sector affiliation) and prior experiences with antimicrobial therapy were used to explore associations with knowledge and behavior-related responses. Chi-square tests were employed to analyze categorical data, specifically to examine the relationship between education level and selected survey responses. Statistical significance was set at α = 0.05.

## RESULTS

### Demographics

**Figure 1:**
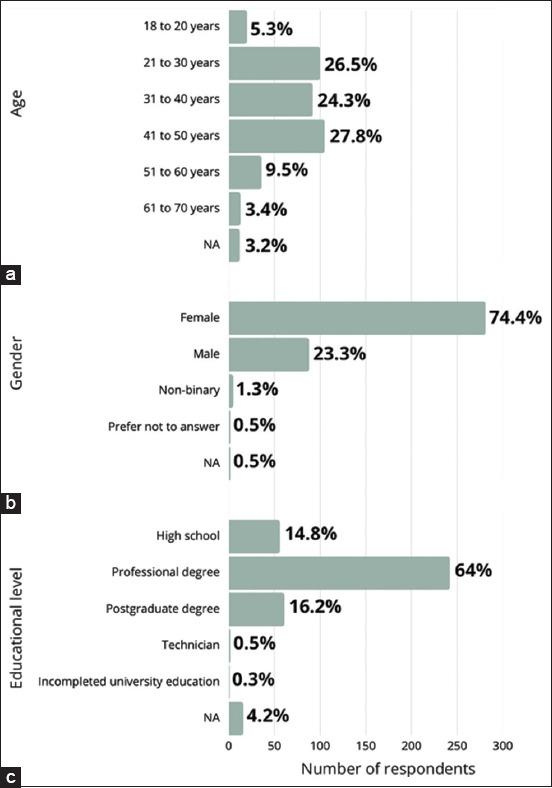
Demographics of dog and cat owners surveyed (n = 378) by (a) age, (b) sex, and (c) highest level of education.

A total of 378 valid responses were collected under Module 1. The most represented age group was 41–50 years (27.8%), followed by 21–30 years (26.5%) and 31–40 years (24.3%) (Figure 1a). The majority of respondents identified as female (74.4%), with male (23.3%) and non-binary individuals (1.3%) comprising the remainder (Figure 1b). In terms of educational attainment, most participants held a professional (bachelor’s) degree (64%), followed by those with postgraduate qualifications (16.2%) and secondary-level education (14.8%). A smaller proportion reported having a technical degree (0.5%), incomplete university studies (0.3%), or did not disclose their education level (4.2%) (Figure 1c). In addition, 35.7% of participants reported working in the human or animal health sector.

### Pet characteristics

**Figure 2:**
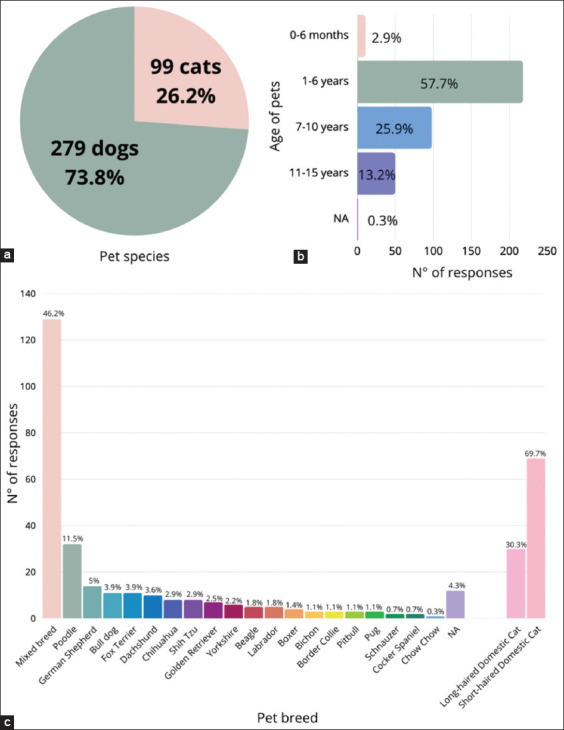
Characterization of the respondents’ pets (n = 378) by (a) pet species, (b) pet age, and (c) pet breed.

According to Module 2, most respondents were dog owners (73.8%), while 26.2% reported owning cats (Figure 2a). The majority of pets were between 1 and 6 years old (57.7%), followed by those aged 7–10 years (25.9%) (Figure 2b). Among dog breeds, mixed breeds were the most common (46.2%), followed by poodles (11.5%) and German shepherds (5%). Among cats, the majority were short-haired domestic cats (69.7%) (Figure 2c).

### Antibiotic knowledge

**Figure 3:**
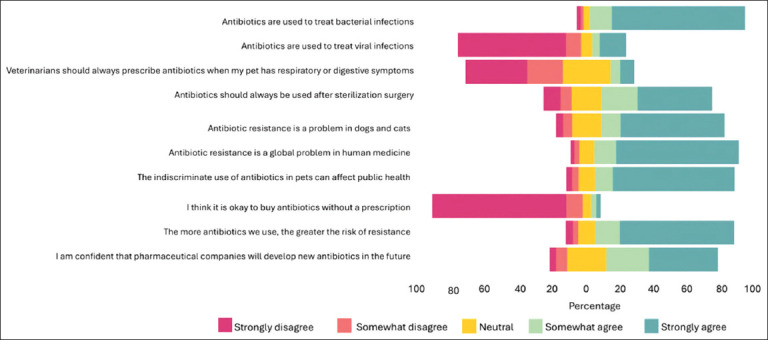
Survey respondents’ general attitudes and knowledge about antibiotics and AMR using a 5-point Likert scale ranging from “strongly agree” to “strongly disagree”.

In Module 3, respondents showed the highest level of agreement with the statement that antibiotics should be used exclusively to treat bacterial infections. The strongest disagreement was noted for the statement suggesting that antibiotics should be obtained without a prescription. The most diverse range of opinions was observed in response to the idea that veterinarians should always prescribe antimicrobials for respiratory or digestive conditions (Figure 3).

### Antimicrobial use behavior

Module 4 captured data on past antibiotic use in pets and owner-veterinarian communication. A significant proportion of respondents (86.2%) reported that their pet had previously received antibiotics. Most participants (82.8%) stated that veterinarians had explained the importance of adhering to prescribed dosage schedules and treatment duration, and 94.2% reported that treatment typically resulted in clinical cure (defined as the resolution of symptoms and return to normal health). However, only 68.2% of respondents indicated that the veterinarian confirmed whether they understood how to administer the antibiotic through the prescribed route (Table 1).

**Table 1:** Opinions on the use of antimicrobials and the veterinarian’s indications for their use.

Question	n	Yes (%)
Has your pet ever received antibiotics?	378	86.2
The veterinarian explained the importance of respecting the administration schedules and treatment duration.	326	82.8
Did the veterinarian ask you if you know how to administer the antibiotics?	326	68.2
Of the times you have used antibiotics on your pet, has the treatment shown clinical cure?	326	94.2

Clarification: N=Total number of people who answered the question; Yes (%): percentage of people who answered “Yes” to the question

Regarding antibiotic use practices, 92.4% of respondents admitted that they would discontinue treatment early if their pet’s condition appeared to improve. In addition, 89% expressed a willingness to pay for diagnostic testing to ensure the correct antibiotic was prescribed. Human antibiotics had been used on pets by 51.9% of respondents, and 38.1% were open to substituting veterinary antibiotics with human medications when feasible. Chi-square analysis showed no statistically significant associations (p < 0.05) between education level and responses to these questions, indicating that education did not influence antibiotic use behaviors (Table 2).

**Table 2:** Association between the highest level of education (high school, professional degree, postgraduate degree, technician, incompleted university education, not answered) and the response to the questions regarding the use of antibiotics.

Question	n	Yes (%)	p-value
If you see improvement in your pet after starting treatment with antibiotics, would you end the treatment sooner than your veterinarian’s prescription?	367	92.4	0.6911
Do you use or have you used human prescription antibiotics on any of your pets?	360	51.9	0.2562
If you could replace a veterinary antibiotic prescribed for your pet with another for human use, would you do it?	373	38.1	0.0920
Would you be willing to pay for an additional examination to determine the most appropriate antibiotic for your pet?	362	89	0.1029

Clarification: N=Total number of people who answered the question; Yes (%): percentage of people who answered “Yes” to the question

In terms of preferred formulations, syrup was the most favored (47.1%) followed by tablets (29.4%). Other preferences included capsules (5.8%), creams or ointments (3.4%), and hospitalization (0.3%). Notably, 13% expressed no preference, and 1.1% did not respond. The most commonly prescribed antibiotic form was tablets (37.6%) followed by syrup (28.8%). Less frequent were injectables (3.1%), capsules (2.9%), and ointments (1.1%). A notable proportion (26.8%) either did not recall the prescribed form or left the question unanswered.

## DISCUSSION

### Owner awareness and understanding of AMR

Bacteria associated with companion animals can develop AMR, much like bacteria affecting humans, particularly when antibiotics are misused in veterinary care [30]. This misuse diminishes treatment efficacy and increases the risk of transmitting resistant strains to humans. Close physical interaction between pets and their owners further exacerbates this risk, underscoring the need for a One Health approach that emphasizes the interconnectedness of human, animal, and environmental health [31, 32].

Pet owners play a pivotal role in mitigating AMR by ensuring the responsible use of antibiotics and maintaining animal health through preventive care [30]. Their behaviors directly influence AMR trends and are essential for both animal welfare and broader public health goals [33, 34]. However, their effectiveness depends heavily on awareness and comprehension of AMR-related issues [35, 36]. Studies by Golovliov *et al*. [36] and Pinto *et al*. [37] show that higher awareness levels are associated with preventive practices that help minimize unnecessary antibiotic use and slow the development of resistance.

In the present study, most respondents demonstrated a basic understanding that antibiotics should be reserved for bacterial infections and acknowledged the importance of AMR as a concern for both human and animal health. Nonetheless, a significant proportion believed antibiotics should always be prescribed for respiratory or digestive symptoms or after surgical procedures such as gonadectomy. This suggests potential misconceptions about when antibiotic treatment is clinically appropriate and may reflect unrealistic expectations that place undue pressure on veterinarians to prescribe medications.

While there was general recognition of the public health risks associated with indiscriminate antibiotic use in pets, the findings suggest only a partial understanding of the One Health framework linking human and animal AMR. Interestingly, many respondents expressed optimism that pharmaceutical companies would develop new antibiotics in the future. Although this outlook is understandable, it may inadvertently reduce the urgency to adopt more conservative antibiotic practices today.

### Demographic comparisons with previous studies

This study found that 74.4% of the 378 respondents were women, which aligns with findings from similar surveys conducted in the United States, Italy, and Australia [19, 23, 24, 38–40]. This may reflect women’s predominant role in making household decisions related to pet care [41]. Alternatively, it could be attributed to higher female participation in face-to-face survey formats, which may introduce sampling bias [42]. Atero *et al*. [43] similarly reported a majority of female respondents in their assessment of Chile’s dog and cat population, suggesting a stronger affinity for companion animals among women.

According to 2023 demographic statistics [44], the percentage of women in Italy, Australia, the U.S., and Chile is around 50%. Specifically, Chile’s 2024 census reported 51.5% female representation [45]. Therefore, the high proportion of female respondents in this study likely reflects gendered interests in animal care rather than demographic imbalance.

Most participants were between 31 and 70 years of age, which is consistent with earlier findings [19, 23, 24, 39–40]. However, only 3.4% of respondents were over 60 years old, in contrast to 30.9% in the study by Atero *et al*. [43]. This discrepancy could be due to the context of data collection, which took place at a large public event likely under-attended by older adults.

With respect to education, most respondents held a professional degree. International comparisons also show a diverse range of educational backgrounds among pet owners. For instance, 41% of Australian respondents had postgraduate education [23], 49% of Italian participants had university degrees [24], and 75% of respondents in another U.S. study held associate degrees or higher [19]. The demographics of this study similarly mirror the findings of Atero *et al*. [43] concerning pet species, age range (1–6 years), and breed distribution, where mixed-breed dogs predominated.

### Communication and adherence in veterinary practice

Addressing AMR effectively requires not only appropriate prescription practices by veterinary professionals but also informed compliance by pet owners [25, 41]. Alarmingly, 92.4% of participants in this study reported that they would stop antibiotic treatment early if their pet appeared to improve – substantially higher than the 17.1% reported by Candellone *et al*. [24]. This behavior suggests significant gaps in understanding proper antibiotic use and may reflect a lack of trust in veterinary guidance.

A growing body of literature supports a shift from paternalistic decision-making models to shared decision-making approaches in veterinary practice, which may help strengthen trust and reduce antibiotic misuse [41]. Veterinarians may also experience pressure from clients to prescribe antibiotics unnecessarily, further complicating stewardship efforts [22, 40].

A study by Marta-Costa *et al*. [46] from Portugal has revealed that even individuals in health-related fields, including undergraduate and postgraduate students, sometimes discontinue pet antibiotic treatments prematurely, highlighting the pervasiveness of knowledge gaps across educational backgrounds.

Economic constraints and the desire for rapid recovery are also common barriers to appropriate antibiotic use [40, 47]. Moreover, “just-in-case” prescribing continues to be a significant contributor to AMR in veterinary settings [48], requiring educational, regulatory, and technological interventions to curb unnecessary prescriptions [49].

In this study, nearly 95% of respondents reported successful clinical recovery following antibiotic treatment, reinforcing the importance of proper use. However, only 68.2% indicated that their veterinarian confirmed whether they fully understood the administration instructions. This highlights a critical opportunity for veterinarians to improve communication and reinforce adherence by providing clear, comprehensive guidance, especially considering that a previous study by Arnecke *et al*. [50] reported high percentages of pet owners’ interest in receiving information regarding AMR.

### Public health implications and One Health considerations

Efforts to reduce antibiotic use in companion animals have largely targeted individual behavior change, focusing either on veterinarians or pet owners. However, a broader and more integrative strategy is needed – one that addresses educational, behavioral, and systemic drivers of AMR [51].

This study enhances the growing understanding of AMR from a One Health perspective by highlighting the often-overlooked role of pet owners in antimicrobial misuse. Key issues identified include the use of human antibiotics on pets and the premature cessation of treatments. These practices increase the risk of resistant pathogens developing in domestic environments and spreading across human-animal-environment interfaces.

Compounding this risk is the fact that some antibiotics used in both humans and animals are also employed in plant agriculture, facilitating the transfer of resistant genes across ecological boundaries [30]. The findings highlight critical gaps in knowledge and practice among pet owners in Chile and emphasize the need for a broader inclusion of veterinary perspectives to develop a cohesive, collaborative, and contextually relevant AMR response.

Moving forward, enhanced public education, stronger veterinarian-client communication, and multisectoral policy development are essential to achieving AMR containment goals. These efforts will help safeguard both animal and human health while advancing the One Health agenda in Chile and beyond.

## CONCLUSION

This study provides critical insight into the perceptions, knowledge, and behaviors of Chilean pet owners regarding antimicrobial use and resistance. Of the 378 respondents surveyed, the majority demonstrated a basic understanding of AMR, particularly that antibiotics should be used exclusively for bacterial infections and not obtained without a prescription. However, misconceptions persisted, many believed antibiotics should be automatically prescribed for respiratory or digestive conditions or post-surgical procedures. Alarmingly, 92.4% indicated they would discontinue treatment early if their pet’s condition improved, and over half (51.9%) had used human antibiotics in animals. Notably, no statistically significant association was found between education level and prudent antimicrobial behaviors, underscoring that awareness gaps exist across demographic strata.

The practical applicability of these findings lies in shaping targeted awareness campaigns and veterinarian communication strategies. Veterinarians should proactively engage pet owners in discussions on treatment adherence, antibiotic specificity, and AMR consequences. Veterinary education and public health messaging should reinforce the One Health perspective, emphasizing the interconnectedness of human, animal, and environmental health and the critical role pet owners play in reducing the spread of resistant bacteria.

A major strength of this study is its pioneering nature, it represents the first survey-based assessment of antimicrobial perceptions among pet owners in Chile, using a validated, multidisciplinary-developed questionnaire administered at a large-scale public pet event. The inclusion of respondents from diverse backgrounds adds valuable baseline data to inform Chile’s national AMR action plan in companion animal care.

However, the study has several limitations. First, the use of convenience sampling at a public pet-focused event may have introduced selection bias, as attendees likely had a greater-than-average interest in animal health. Second, geographic coverage was limited to Santiago, where access to veterinary services is relatively high; thus, the findings may not fully reflect the perceptions of rural or underserved populations. Third, the cross-sectional nature of the study restricts causal inference and only captures a snapshot in time. In addition, veterinary perspectives were not captured, limiting the ability to triangulate owner-reported data with professional practice.

Future research should expand geographically to include rural regions and underrepresented populations and incorporate veterinarian and policy-maker perspectives for a more comprehensive understanding. Longitudinal studies could also help assess how interventions influence owner knowledge and behavior over time. In addition, qualitative methods such as interviews or focus groups may provide a richer context to further explore motivational drivers and barriers to antimicrobial stewardship among pet owners.

This study highlights significant gaps in antimicrobial use behaviors among Chilean pet owners, despite overall awareness of AMR. These findings emphasize the need for improved veterinary-client communication, stronger public education campaigns, and integrative One Health policy strategies. By leveraging the central role of pet owners in antimicrobial administration, Chile has the opportunity to enhance its national AMR response and contribute meaningfully to global efforts in preserving antibiotic efficacy.

## AUTHORS’ CONTRIBUTIONS

NG and AD: Writing – original draft, and writing – review and editing. PT: Formal analysis, project administration, investigation, methodology, and writing – review and editing. JL: Methodology, data analysis, and data curation. LCR: Data obtention and writing – original draft. AN: Data acquisition; BG: Data analysis. All authors have read and approved the final manuscript.

## References

[1] Founou L.L, Founou R.C, Essack S.Y (2021). Antimicrobial resistance in the farm-to-plate continuum:More than a food safety issue. Future Sci. OA.

[2] Aslam B, Asghar R, Muzammil S, Shafique M, Siddique A.B, Khurshid M, Ijaz M, Rasool M.H, Chaudhry T.H, Aamir A, Baloch Z (2024). AMR and sustainable development goals:At a crossroads. Global Health.

[3] Angeles Flores G, Cusumano G, Venanzoni R, Angelini P (2025). Advancements in antibacterial therapy:Feature papers. Microorganisms.

[4] Mullard A (2016). An audience with. Jim O'Neill. Nat. Rev. Drug Discov.

[5] Ahmed S.M, Naher N, Tune S.N.B.K, Islam B.Z (2022). The implementation of national action plan (NAP) on antimicrobial resistance (AMR) in Bangladesh:Challenges and lessons learned from a cross-sectional qualitative study. Antibiotics (*Basel*).

[6] WHO (2015). Global Action Plan on Antibiotic Resistance. Available from:https://iris.who.int/bitstream/handle/10665/193736/9789241509763_eng.pdf?sequence=1.

[7] Ranjalkar J, Chandy S.J, Sartelli M, Coccolini F, Catena F, Pagani L (2024). One Health and antimicrobial resistance. Global Infection Prevention and Management in Healthcare. Antimicrobial resistance and One Health. Advanced Meeting Solutions.

[8] Jakhar A.M, Faqir Y, Jakhar K.A, Abro F.A, Bhart Ma, J, Kumar P, Dubey R.C (2025). Impact of nanofertilizers on human/animal health and ecosystem. Nanofertilizers for Sustainable Agriculture:Assessing Impacts on Health, Environment, and Economy.

[9] Vezeau N, Kahn L.H, Sander W.E (2025). Antimicrobial resistance–a one health issue. Global One Health and Infectious Diseases.

[10] Guardabassi L, Schwarz S, Lloyd D.H (2004). Pet animals as reservoirs of antimicrobial-resistant bacteria. J. Antimicrob. Chemother.

[11] Pomba C, Rantala M, Greko C, Baptiste K.E, Catry B, Van Duijkeren E, Törneke K (2017). Public health risk of antimicrobial resistance transfer from companion animals. J. Antimicrob. Chemother.

[12] Caneschi A, Bardhi A, Barbarossa A, Zaghini A (2023). The use of antibiotics and antimicrobial resistance in veterinary medicine, a complex phenomenon:A narrative review. Antibiotics (*Basel*).

[13] Wijayanti A.D, Rosetyadewi A.W, Pratama A.M, Septana A.I, Setyawan D.C.B, Fitriana I (2023). A recent update on the use of antimicrobials for animal health in Yogyakarta, Indonesia. Int. J. One Health.

[14] Wayne A, McCarthy R, Lindenmayer J (2011). Therapeutic antibiotic use patterns in dogs:Observations from a veterinary teaching hospital. J. Small Anim. Pract.

[15] Buckland E.L, O'Neill D, Summers J, Mateus A, Church D, Redmond L, Brodbelt D (2016). Characterisation of antimicrobial usage in cats and dogs attending UK primary care companion animal veterinary practices. Vet. Rec.

[16] Van Cleven A, Sarrazin S, de Rooster H, Paepe D, Van der Meeren S, Dewulf J (2018). Antimicrobial prescribing behaviour in dogs and cats by Belgian veterinarians. Vet. Rec.

[17] Mateus A.L.P, Brodbelt D.C, Barber N, Stärk K.D.C (2014). Qualitative study of factors associated with antimicrobial usage in seven small animal veterinary practices in the UK. Prev. Vet. Med.

[18] Scarborough R.O, Sri A.E, Browning G.F, Hardefeldt L.Y, Bailey K.E (2023). “Brave enough”:A qualitative study of veterinary decisions to withhold or delay antimicrobial treatment in pets. Antibiotics (*Basel*).

[19] Taylor D.D, Scallan Walter E.J (2023). Colorado pet owners'perceptions of and attitudes towards antimicrobial drug use and resistance. Vet. Rec.

[20] Frey E, Kedrowicz A, Hedgpeth M.W (2023). Decision making on antimicrobial use:Cat and dog owners'knowledge and preferences for veterinary communication. Vet Rec.

[21] King C, Smith M, Currie K, Dickson A, Smith F, Davis M, Flowers P (2018). Exploring the behavioural drivers of veterinary surgeon antibiotic prescribing:A qualitative study of companion animal veterinary surgeons in the UK. BMC Vet. Res.

[22] Smith M, King C, Davis M, Dickson A, Park J, Smith F, Flowers P (2018). Pet owner and vet interactions:Exploring the drivers of AMR. Antimicrob. Resist. Infect. Control.

[23] Scarborough R, Hardefeldt L, Browning G, Bailey K (2021). Pet owners and antibiotics:Knowledge, opinions, expectations, and communication preferences. Antibiotics (*Basel*).

[24] Candellone A, Badino P, Girolami F, Ala U, Mina F, Odore R (2023). Dog owners'attitude toward veterinary antibiotic use and antibiotic resistance with a focus on canine diarrhea management. Animals (*Basel*).

[25] Redding L.E, Cole S.D (2019). Pet owners'knowledge of and attitudes toward the judicious use of antimicrobials for companion animals. J. Am. Vet. Med. Assoc.

[26] Ministerio de Salud de Chile (2017). Plan Nacional Contra la Resistencia a los Antimicrobianos. Available from:https://diprece.minsal.cl/wrdprss_minsal/wp-content/uploads/2017/08/plan-nacional-contra-la-resistencia-a-los-antimicrobianos.pdf.

[27] Sandoval J, Moraes dos Santos F, Romero J.F (2024). National data on antimicrobial resistance (AMR) in Chile in the context of community-acquired respiratory tract infections:Links between antimicrobial susceptibility, local and international antimicrobial prescribing guidelines, access to medicines, and clinical outcomes. Rev. Chil. Infectol.

[28] Cabrera-Pardo J.R, Lood R, Udekwu K, Gonzalez-Rocha G, Munita J.M, Järhult J.D, Opazo-Capurro A (2019). A One Health-One World initiative to control antibiotic resistance:A Chile-Sweden collaboration. One Health.

[29] Galarce N, Arriagada G, Sánchez F, Venegas V, Cornejo J, Lapierre L (2021). Antimicrobial use in companion animals:Assessing veterinarians'prescription patterns through the first national survey in Chile. Animals (*Basel*).

[30] Velazquez-Meza M.E, Galarde-López M, Carrillo-Quiróz B, Alpuche-Aranda C.M (2022). Antimicrobial resistance:One health approach. Vet. World.

[31] Robinson T.P, Bu D.P, Carrique-Mas J, Fèvre E.M, Gilbert M, Grace D, Woolhouse M.E (2016). Antibiotic resistance is the quintessential One Health issue. Trans. R. Soc. Trop. Med. Hyg.

[32] Joosten P, Ceccarelli D, Odent E, Sarrazin S, Graveland H, Van Gompel L, Battisti A, Caprioli A, Franco A, Wagenaar J.A, Mevius D, Dewulf J (2020). Antimicrobial usage and resistance in companion animals:A cross-sectional study in three European countries. Antibiotics (*Basel*).

[33] Overgaauw P.A.M, Vinke C.M, van Hagen M.A.E, Lipman L.J.A (2020). A one health perspective on the human–companion animal relationship with emphasis on zoonotic aspects. Int. J. Environ. Res. Public Health.

[34] Vercelli C, Gambino G, Amadori M, Re G (2022). Implications of Veterinary Medicine in the comprehension and stewardship of antimicrobial resistance phenomenon. From the origin till nowadays. Vet. Anim. Sci.

[35] McEwen S.A, Collignon P.J, Schwarz S, Cavaco L.M, Shen J, Møller Aarestrup F (2018). Antimicrobial resistance:A one health perspective. Antimicrobial Resistance in Bacteria from Livestock and Companion Animals.

[36] Golovliov K, León D, Silva P, Falcón N (2021). Medication without veterinary prescription in pets in Lima, Peru (2020). Rev. Investig. Vet. Perú.

[37] Pinto J.C, Keestra S, Tandon P, Chandler C.I (2020). WASH and biosecurity interventions for reducing burdens of infection, antibiotic use and antimicrobial resistance in animal agricultural settings:A One Health mixed methods systematic review. Lancet Planet Health.

[38] Slater M.R, Di Nardo A, Pediconi O, Dalla Villa P, Candeloro L, Alessandrini B, Del Papa S (2008). Cat and dog ownership and management patterns in central Italy. Prev. Vet. Med.

[39] Vinassa M, Vergnano D, Valle E, Giribaldi M, Nery J, Prola L, Bergero D, Schiavone A (2020). Profiling Italian cat and dog owners'perceptions of pet food quality traits. BMC Vet. Res.

[40] Cazer C.L, Lawless J.W, Frye A, Gonzalez L, Safi A.G ((2023a)). Divergent veterinarian and cat owner perspectives are barriers to reducing the use of cefovecin in cats. J. Am. Vet. Med. Assoc.

[41] Janke N, Coe J.B, Bernardo T.M, Dewey C.E, Stone E.A (2021). Pet owners'and veterinarians'perceptions of information exchange and clinical decision-making in companion animal practice. PLoS One.

[42] Gates M, Walker J, Zito S, Dale A (2019). Cross-sectional survey of pet ownership, veterinary service utilisation, and pet-related expenditures in New Zealand. N. Z. Vet. J.

[43] Atero N, Córdova-Bührle F, Salgado-Caxito M, Benavides J.A, Fernández M, Diethelm-Varela B, Ramos R, Aguirre C.P, Trujillo F, Dürr S, Mardones F.O (2024). An assessment of the owned canine and feline demographics in Chile:registration, sterilization, and unsupervised roaming indicators. Prev. Vet. Med.

[44] Datosmacro.com Demografía. Población.

[45] Instituto Nacional de Estadísticas de Chile. Estadísticas. Censos de Población y Vivienda.

[46] Marta-Costa A, Miranda C, Silva V, Silva A, Martins Â, Pereira J.E, Maltez L, Capita R, Alonso-Calleja C, Igrejas G, Poeta P (2021). Survey of the knowledge and use of antibiotics among medical and veterinary health professionals and students in Portugal. Int. J. Environ. Res. Public Health.

[47] Lavigne S.H, Louis S, Rankin S.C, Zaoutis T.E, Szymczak J.E (2021). How companion animal veterinarians in the United States perceive financial constraints on antibiotic decision-making. Vet. Rec.

[48] Currie K, King C, Nuttall T, Smith M, Flowers P (2018). Expert consensus regarding drivers of antimicrobial stewardship in companion animal veterinary practice:A Delphi study. Vet. Rec.

[49] Cazer C.L, Frye A, Lawless J.W, Gonzalez L, Cobo-Angel C, Safi A.G ((2023b)). Pathways to sustainable antimicrobial use in cats. J. Am. Vet. Med. Assoc.

[50] Arnecke A.L, Schwarz S, Lübke-Becker A, Jensen K.C, Bahramsoltani M (2024). A survey on companion animal owners'perception of veterinarians'communication about zoonoses and antimicrobial resistance in Germany. Animals (*Basel*).

[51] Tompson A.C, Mateus A.L, Brodbelt D.C, Chandler C.I (2021). Understanding antibiotic use in companion animals:A literature review identifying avenues for future efforts. Front. Vet. Sci.

